# A Genetic Analysis of Current Medication Use in the UK Biobank

**DOI:** 10.3390/jpm14030319

**Published:** 2024-03-20

**Authors:** Palle Duun Rohde

**Affiliations:** Genomic Medicine, Department of Health Science and Technology, Aalborg University, 9220 Aalborg, Denmark; palledr@hst.aau.dk

**Keywords:** GWAS, polygenic prediction, genomic medicine, complex traits

## Abstract

Genomics has been forecasted to revolutionise human health by improving medical treatment through a better understanding of the molecular mechanisms of human diseases. Despite great successes of the last decade’s genome-wide association studies (GWAS), the results have been translated to genomic medicine to a limited extent. One route to get closer to improved medical treatment could be by understanding the genetics of medication use. Current medication profiles from 335,744 individuals from the UK Biobank were obtained, and a GWAS was conducted to identify common genetic variants associated with current medication use. In total, 59 independent loci were identified for medication use, and approximately 18% of the total variation was attributable to common genetic variation. The largest fraction of genetic variance for current medication use was captured by variants with low-to-medium minor allele frequency, with coding, conserved genomic regions and transcription start sites being enriched for associated variants. The average correlation (R) between medication use and the polygenic score was 0.14. The results further demonstrated that individuals with higher polygenic burden for medication use were, on average, sicker and had a higher risk for adverse drug reactions. These results provide an insight into the genetic contribution of medication use and pave the way for developments of novel multiple trait polygenic scores, which include the genetically informed medication use.

## 1. Introduction

Understanding the relationship between DNA sequence variation and the predisposition to common diseases has interested researchers for decades. In particular, after the initial release of the human genome [1], the number of polymorphic genetic variants associated with disease predisposition has grown exponentially to more than 60,000 associations [2,3,4]. Genome-wide association studies (GWAS) have provided new insight into the biology and genetic epidemiology of many human complex diseases, which is essential for innovative developments within genomic medicine.

Genomic medicine aims to develop treatment approaches based on the individual’s genetic makeup, environmental exposures and lifestyle parameters [5,6], and it is foreseen to change the way we prevent, diagnose and treat medical conditions. Fundamental to the development of genomic medicine is accurate knowledge regarding the disease pathogenesis and acknowledging the genetic contributions to variation in how patients respond to treatment [7]. Genetic variation among patients modulates drug efficiency and can impose toxic effects (adverse drug reactions) [8]; thus, understanding how genetic variation affects drug response is essential for the development of genomic medicine.

A major challenge and hindrance in studying the genetic factors influencing drug response variability is the lack of accessible data. Despite the emergence of large biobanks, such as the United Kingdom Biobank [9], Japan Biobank [10] and Estonia Biobank [11], which contain genetic and deep phenotypic information on the participants, information on response to medical treatment is absent. The accessibility of electronic health records and self-reported health status may provide means to alternative approaches for studying the genetic basis of traits of relevance for medication use.

Previously, Wu et al. [12] performed a genetic analysis of medication use in the United Kingdom Biobank (UKB). They categorised medications based on the drugs’ active substances according to the organ or system they act on and their pharmacological properties. They performed a genetic analysis of 23 isolated groups of medications and identified a very large number of independent loci associated with the different drug categories. The aim of the current study was to investigate the genetic basis of self-reported medication use in the UKB. Current medication use was defined as the total number of different prescription and over-the-counter medications UKB participants were taking at the time of the first verbal interview. Medication use—as defined above—has a clear advantage, in that it is easily quantifiable compared to, for example, drug efficiency. Assuming current medication use as a quantitative trait phenotype, a genetic analysis of 335,744 unrelated individuals from the UKB was conducted. The hypothesis was that across medical conditions, medication use has a detectable genetic component, and medication use was expected to be genetically correlated with common diseases, as commonly prescribed drugs are likely to serve as proxy phenotypes for major disease groups. Extensive medication use among individuals older than 65 years has been shown to be associated with ill health and morbidity [13,14]; hence, it was further hypothesised that medication use was genetically correlated with health-related outcomes. If medication use has a genetic component, understanding the genetic basis is important because many medications have side effects, and increased drug usage might be associated with higher risk of toxic effects. Hence, genetic predisposition to high medication use could be used as guidance in treatment plans aiming to reduce the total number of medications.

## 2. Materials and Methods

### 2.1. Genotype and Phenotype Data

Genetic and phenotypic data were obtained from the United Kingdom Biobank (UKB) [9]. Data were collected for more than 500,000 individuals aged 37–73 years. Details on how chip genotyping was performed were described previously [9]. In order to obtain a genetically homogeneous study population, the analyses were restricted to unrelated Caucasians (Data Fields: 22013, 21000, 22006) and excluded individuals with more than 5000 missing genotypes or individuals with autosomal aneuploidy, resulting in a study population size of 335,744 individuals. The chip genotype was imputed using the Haplotype Reference Consortium (HRC) and UK10K haplotype resource, as described by Bycroft et al. [9]. The imputed genotype probabilities were converted to hard-call genotypes using PLINK2 (hard call: 0.1) [15]. Genetic variants with minor allele frequency (MAF) < 0.01, missing genotype rate > 0.05, Hardy–Weinberg equilibrium test *p*-value < 1 × 10^−6^ or imputation info score < 0.3 were excluded, resulting in a total of 9,804,629 SNPs left for analysis.

Current medication use was defined as the number of different prescription and over-the-counter medicines (Data Field: 20003) the participants were taking regularly at the time of the verbal interview. Only phenotypic information from the initial assessment (conducted between 2006 and 2010) was included in the analysis, as the first instance contained the lowest number of non-missing samples. Any short-term medications, such as antibiotics or analgesics, were not registered at the interview. A list of ICD10 codes commonly used to describe adverse drug reactions was obtained from Hohl et al. [16] (Supplementary Materials: Table S1).

### 2.2. Genome-Wide Association Study (GWAS) of Medication Use

The 9,804,629 SNPs remaining after initial quality control were used to conduct a GWAS on medication use within the entire White British cohort (335,744 individuals) using PLINK2 software [15]. Sex, age, UKB assessment centre and the first ten genetic principal components (Data Field: 22009) were included as covariates in the GWAS. To identify high-confidence independent associated loci, LD-based clumping was performed with a window size of 1000 kb with r^2^ < 0.01, and for the major histocompatibility complex region, only one significant locus was allowed. In line with previous work [17,18], the lead SNP within each independent genome-wide significant locus was annotated to the nearest gene (genome build GRCh37, hg19) within 2000 kb using Variant Effect Predictor [19].

### 2.3. Estimation of Heritability and Genetic Correlations

The proportion of variation in medication use explained by common SNPs ($h_{SNP}^{2}$) was estimated using SumHer [20]. Genetic variants within the HLA region were excluded prior to analysis, as suggested by the authors of SumHer [20], and estimation was performed assuming the LDAK heritability model. In addition, heritability enrichment across 24 functional categories was estimated (obtained from Finucane et al. [21]). Moreover, the $h_{SNP}^{2}$ was partitioned to autosomal chromosomes and minor allele frequency bins.

As it has been shown that SumHer and LD Score regression [22] had similar accuracies in the estimation of genetic correlations [20], LD Hub was used to estimate the genetic correlations between medication use (excluding the HLA region) and 257 quantitative and disease traits [23]. To account for multiple testing, all *p*-values were adjusted with a Bonferroni correction (*P*_adj_ < 0.01). Using LD Score regression [22], the genetic correlation between medication use and the previously published genetic analysis of categories of medication traits [12] was computed. The univariate LD scores were computed using the 1000 Genomes European data.

### 2.4. Polygenic Scores for Medication Use

The genetic burden for medication use was computed using polygenic scores (PGS). First, the White British UKB cohort was divided into five equally sized parts. Then, five new GWASs (using the same covariates as described above) were conducted, removing one-fifth of the samples every time (i.e., a five-fold cross-validation scheme). For each of the five sets of GWAS summary statistics, LD clumping was performed using different LD cut-offs (r^2^ < {0.1, 0.3, 0.5, 0.7, 0.9}) for a range of *p*-value thresholds (*p* < 0.001, 0.01, 0.05, 0.1, 0.2, 0.3, 0.4, 0.5, 0.7, 0.9, 0.999).

For each of the five GWAS summary statistics, the polygenic score for the one-fifth of the samples not included in the GWAS was computed as $PGS=\sum_{i=1}^{m}w_{i}{\hat{b}}_{i}$, where $w_{i}$ is the *i*-th genotype (allelic counts); ${\hat{b}}_{i}$ is the estimated GWAS SNP effect; and m is the number of SNPs left after LD pruning and *p*-value thresholding. LD clumping and thresholding and computation of polygenic scores were performed in the R package qgg [24,25].

The accuracy of polygenic scores was obtained as the average correlation between the number of medications taken by individuals in the validation set (i.e., the one-fifth of the samples not included in the GWAS) and the computed polygenic score for the same individuals. Polygenic scores were divided into percentiles, and regression coefficients (β) were estimated using linear regression of the number of medications taken in the polygenic score percentile relative to the 50th polygenic score percentile, adjusted for sex, age, UKB assessment centre and the first ten genetic principal components.

## 3. Results

This study presents the results of a genetic analysis of current medication use within the White British cohort from the UK Biobank (*n* = 335,744). Current medication use was defined as the number of different prescription and over-the-counter medicines the participants were taking regularly at the time of the verbal interview (short-term medications, such as antibiotics or analgesics, were not included). The average number of medications taken by males was 2.34 (standard deviation (SD 2.7) and 2.67 (SD 2.7) for females), with a linear increase in the number of medications taken with increasing age (Supplementary Materials: Figure S1). Interestingly, the mean number of drugs taken by individuals with an ICD10 code for adverse drug reaction (Supplementary Materials: Table S1) [16] was significant larger (mean = 4.09, SD = 3.57) than the mean number of drugs taken by individuals without such diagnoses (mean = 2.23, SD = 2.43; *t*-test = −101.77, df = 46,662, *p* < 2.2 × 10^−16^), suggesting that individuals taking a larger number of medications are more likely to encounter an adverse drug reaction or that individuals experiencing adverse drug reactions are more difficult to treat, requiring more medication. Participants reported a total of 3247 different medications, where the most frequently used drugs were paracetamol (*n* = 61,604), aspirin (*n* = 44,894), ibuprofen (*n* = 41,756) and simvastatin (*n* = 38,379).

**Figure 1:**
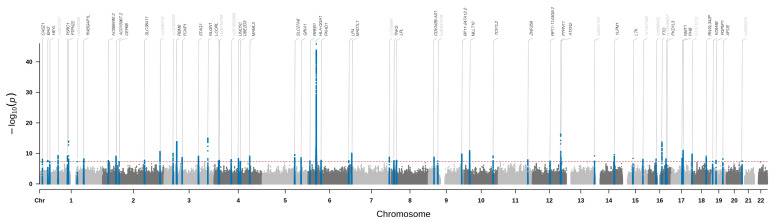
Manhattan plot of medication use in the UKB (*n* = 335,744). The x-axis is the chromosomal position, and the y-axis shows the negative logarithm base-10 for the *p*-values from regression of current medication use from 9,804,629 SNPs. The horizontal red line shows the genome-wide significance level (5 × 10^−8^). Independent genome-wide significant loci (within 1000 kb and r^2^ < 0.01) are depicted in blue. For each significant locus, the gene within 2000 kb is shown (for intergenic loci, the lead SNP is shown).

After SNP quality control (see the Materials and Methods section), there were 9,804,629 autosomal SNPs left for GWAS analysis. In total, 59 independent quantitative trait loci for current medication use were identified (Figure 1, Supplementary Materials: Table S2). The strongest associated locus was found within the human leucocyte antigens (HLA) complex (rs35248896, *p* = 1.52 × 10^−46^). Because of the complexity of the HLA region [26,27], only one significant locus at this genomic region was allowed (additional three loci passed the significance threshold within the HLA region but were excluded; Supplementary Materials: Table S2). Among the 59 genome-wide associated loci, 14 of them were located in intergenic regions.

**Figure 2:**
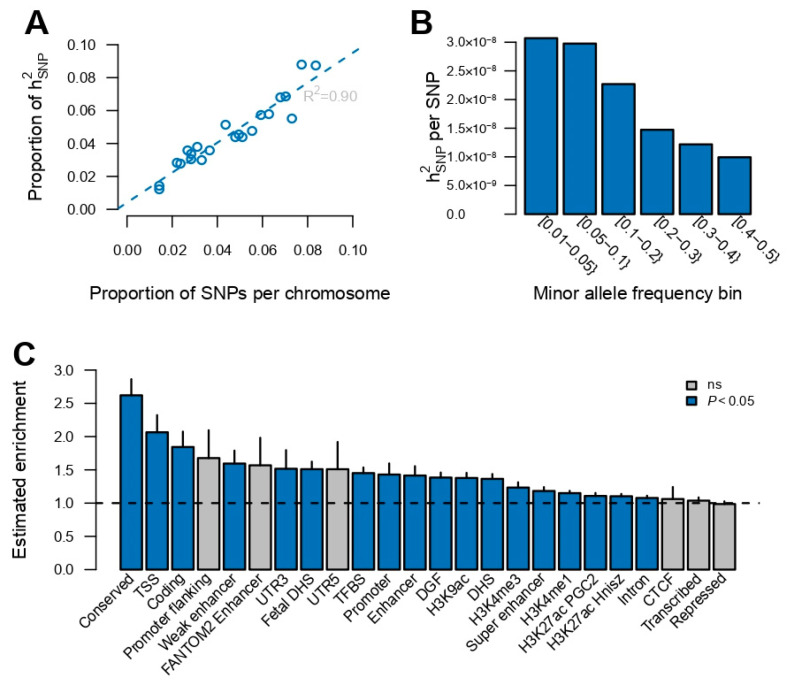
Partitioning of genomic variance for medication use. (**A**) Proportion of genomic variance captured per autosomal chromosome as function of the proportion of SNPs per chromosome. (**B**) Proportion of genomic variance, scaled by the number of SNPs, captured by minor allele frequency. (**C**) Estimated enrichment score for functional categories. Vertical line segments mark the standard deviation of the enrichment score. Horizontal dashed line marks an enrichment score of no enrichment. CTCF: a highly conserved multifunctional DNA-binding protein, DGF: digital genomic footprint, DHS: DNase I hypersensitivity sites, TFBS: transcription factor binding site, TSS: transcription start site.

Using the medication use GWAS summary statistics (excluding the HLA region), the proportion of variation in medication use explained by the SNPs ($h_{SNP}^{2}$) was estimated as 0.18 ± 0.005. Next, the total heritability was partitioned to the heritability captured by individual autosomal chromosomes, and a linear association was found between the proportion of heritability captured by each autosomal chromosome and the number of SNPs per chromosome (R^2^ = 0.9, Figure 2A), suggesting that medication use is a highly polygenic trait. The genomic variance explained per variant within minor allele frequency bins indicated that low-frequency variants captured about three times more genetic variance than high-frequency variants (Figure 2B). Additionally, an enrichment score across 24 functional categories was computed (obtained from Finucane et al. [21]), which is the estimated share of $h_{SNP}^{2}$ divided by its expected share under the assumed heritability model [20,21] (Figure 2C). In particular, the conserved genomic region and transcription start sites (TSS) were highly enriched, accounting for 4.5% and 1.3% of $h_{SNP}^{2}$, respectively.

**Figure 3:**
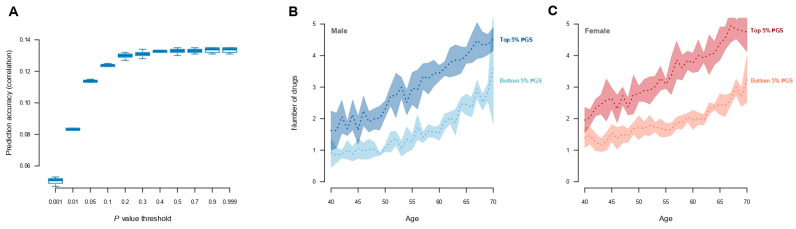
Prediction of the number of medications used. (**A**) Prediction accuracy (measured by the correlation between observed and predicted values) for medication use across the range of *p*-values. The results are shown for r^2^ < 0.5, as it gave the highest accuracy across different r^2^ values (see Supplementary Materials: Figure S2 for all r^2^ values). (**B**,**C**) Averaged number of drugs (over the five training sets) used by males and females stratified by top 5% and bottom 5% of polygenic scores. Shading corresponds to the standard error over the five training sets.

Polygenic scores for medication use were constructed by re-estimating the SNP effects using a five-fold cross-validation scheme. The scoring was performed on five levels of LD pruning (r^2^) and across eleven *p*-value thresholds. The maximum prediction accuracy (Pearson’s correlation, R, between the polygenic scores and current medication use ~0.14) was obtained when markers with r^2^ > 0.5 (Supplementary Materials: Figure S2) were removed at a *p*-value of 0.9 (Figure 3A), which included approximately 1.5 million genetic markers (Supplementary Materials: Figure S3). By stratifying individuals based on their polygenic score, the individuals within the top 5% highest polygenic scores had increased medication use compared with individuals with the 5% lowest polygenic scores (Figure 3B, Supplementary Materials: Figure S4). Moreover, individuals with the 5% highest polygenic scores had significantly more ICD10 diagnoses than those individuals with 5% lowest polygenic scores (10.8 diagnoses and 6.4 diagnoses, respectively; Supplementary Materials: Figure S5). There was, however, no visual difference with regard to the diseases they were diagnosed with (Supplementary Materials: Figure S6); individuals with the highest polygenic scores for medication use simply had more diagnoses than those with low polygenic scores (Supplementary Materials: Figures S5B and S6). Using the classification of adverse drug reactions from Hohl et al. [16] (Supplementary Materials: Table S1), it could be seen that those individuals with high polygenic scores who experienced adverse drug reactions had, on average, a 1.6-fold higher medication use compared with low-risk individuals (Supplementary Materials: Figure S7 and Table S4).

**Figure 4:**
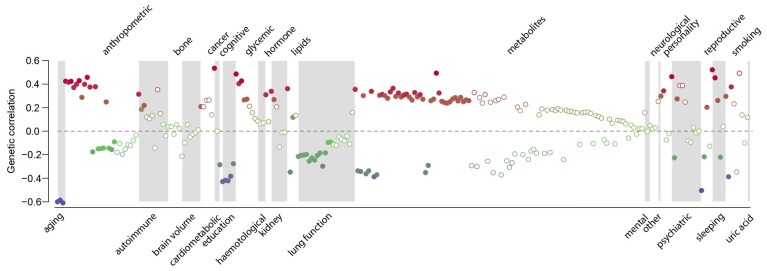
Estimated genetic correlations between medication use and 257 traits and diseases. Traits displaying significant genetic correlations are displayed as filled symbols. The colour indicate the magnitude of the estimated genetic correlation. Details can be found in Supplementary Materials: Table S3.

**Figure 5:**
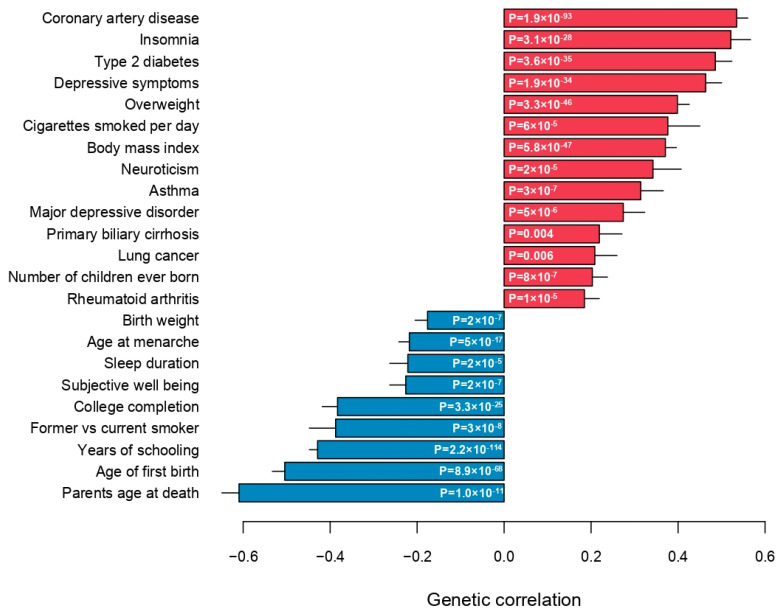
Genetic correlations between medication use and selected top categories.

Finally, the genetic correlations between medication use and 257 quantitative traits and complex diseases were computed using LD Hub [23]. Significant genetic correlations with 115 traits (Bonferroni-adjusted *p*-value < 0.01) across 26 categories were identified, except bone traits, where no genetic correlation with medication use was found (Figure 4, Supplementary Materials: Table S3). As expected, medication use was positively genetically correlated with major common complex diseases, in particular coronary artery disease, type 2 diabetes, asthma, lung cancer and major depressive disorder (Figure 5). Parents’ age at death was the trait, which was most negatively genetically correlated with medication use, indicating that higher medication use correlated with lower age at death (higher mortality) of the parents (Figure 5). The number of years in school and completion of college education were negatively correlated with medication use. The number of cigarettes smoked per day and medication use were positively genetically correlated, and medication use was also genetically correlated with sleep traits, such as insomnia and sleep duration (Figure 5). Finally, the genetic correlations between medication use and the medication categories previously published by Wu et al. [12] were estimated (Supplementary Materials: Figure S8). The average genetic correlation between our definition of medication use and the 23 medication categories was 0.52 (SD 0.22; Supplementary Materials: Table S5), and the category ‘drugs affecting bone structure and mineralization’ was the only insignificant result, which agreed with the observation that medication use was not genetically correlated with any bone traits (Figure 4).

## 4. Discussion

In this study, data from UKB were used to perform a genome-wide genetic analysis of current medication use, defined as the total number of different prescription and over-the-counter drugs the participants from UKB were taking at the time of the initial assessment. The aim was to investigate the genetic basis of self-reported medication use. A total of 59 linkage disequilibrium independent SNPs (*p* < 5 × 10^−8^) associated with current medication use were identified. The strongest statistical signal was located within the major histocompatibility complex (MHC); HLA-DQA1 (lead SNP rs35248896, *p*-value = 1.52 × 10^−46^), which belongs to the MHC class II gene. The MHC region is a large genomic region on chromosome 6, which is associated with more diseases than any other region of the genome [27,28]. Additional three loci within MCH reached LD-independent genome-wide significance (Supplementary Materials: Table S2); however, the complexity and extreme variant polymorphism, combined with strong LD within MHC, complicate the interpretation and disentanglement of individual MHC loci [29]. Given the biological involvement of MHC in immune response, it was unsurprising that this precise genomic region contained the strongest associated loci for current medication use.

The genome-wide associated loci have previously been linked to a large number of different quantitative traits and multifactorial complex diseases. Since genetic correlations express the extent to which two quantitative phenotypes reflect what is genetically the same character [30], it was not surprising to observe good correspondence between the identified genome-wide associated genomic loci and their previous associations and significant genetic correlations. For example, among the candidate genes were known susceptibility loci for diabetes (*PTPN22*, *CEP68*, *RREB1*, *TCF7L2* [31]), coronary artery disease (*PSRC1*, *UNC5C*, *LPLA* [32]), depression (*MAD1L1*, *YLPM1* [33,34]) and insomnia (*NMT1* [35]). Genes previously associated with non-disease traits, including BMI (*RABGAP1L*, *HEYL* [36,37]), smoking (*NLGN1* [38]) and age at menarche (*RBM6* [39]), were also among the associated loci.

The polygenic nature of medication use was—aside from the large number of identified quantitative trait loci—further supported by the linear association between the proportion of genetic variance captured by each autosomal chromosome and the proportion of genetic variants located on each chromosome, which was similar to what is observed for other polygenic traits [25,40,41,42,43]. Low-frequency genetic variants captured more genetic variance than common genetic variants, which was similar to what is observed in, for example, type 2 diabetes [44] and coronary artery disease [42,45]. Neuro-developmental and -degenerative disorders, such as schizophrenia, Tourette syndrome and Alzheimer’s disease, do, however, show the opposite pattern [40,41,46]. Therefore, it is not surprising that the findings correspond with what is observed for common diseases, since the disease prevalence of common diseases in the UKB follows population prevalence; furthermore, the prevalence of mental disorders is too low compared to population frequency.

The statistical genetic analysis was performed across all in-hospital medical conditions the UKB participants may have been diagnosed with prior to the initial assessment between 2006 and 2010. The focus of the present study was on studying the genetic contributions to variation in current medication use. Thus, the results of the genetic association could be biased towards common diseases with the highest disease prevalence, for example, through partially shared genetic aetiology. However, this would inevitably imply that some disease groups require numerically more drugs for treatment than other disease groups. Moreover, medication use was also strongly genetically correlated with complex traits, such as smoking behaviour, parents’ age at death, educational level and insomnia, suggesting that the genetic architecture of medication use was not per se biased towards common diseases. Clearly, the statistical genetic findings presented in the current study should be validated in future studies. Other large biobank projects, such as Japan Biobank [10] or Estonia Biobank [11], could serve as valuable replication cohorts, although they are subject to other selection and recruitment biases than the UK Biobank. Moreover, as discussed below, inaccurate trait definitions increase phenotypic heterogeneity among cohorts, which reduces statistical power and diminishes the prediction accuracy of polygenic predictions [47,48]; therefore, these should be taken into consideration when performing any genetic replication.

The results presented demonstrated that individuals with high polygenic scores for medication use had an increased medication usage, although the degree of variance explained by polygenic scores remained very moderate. The increased polygenic burden for medication use was associated with higher probability of being diagnosed with multiple diseases and also having experienced adverse drug reactions. Therefore, this presents an opportunity for future applications, wherein the polygenic score associated with medication usage could potentially serve as a means of identifying individuals at elevated risk. Specifically, individuals with the highest polygenic scores for medication usage could be targeted for heightened medical attention to mitigate the occurrence of adverse drug reactions. This proactive approach holds promise in optimising medication management and enhancing patient safety by tailoring medical interventions to individuals’ genetic predispositions. Medication use was genetically correlated with known indicators of poor health. For example, overweight and high body mass index—known to be strongly genetically influenced [49]—were positively correlated with medication use, and these are strongly associated with poor health [50]. Moreover, the behavioural characteristics of smoking and sleep patterns are also known factors for bad state of health [51], and they also exhibited significant genetic correlations with medication use.

The results clearly demonstrated a link between the polygenic burden for the number of different medications being used by the individual and the individual’s overall health status. These findings could have future clinical applications within precision medicine initiatives, as the current—and potentially future—medication profile is predictive of an individual’s future health status. Polygenic scores play a crucial role in customising preventive measures and treatments based on an individual’s genetic risk profile, leading to notable enhancements in patient outcomes and healthcare efficiency. Furthermore, the incorporation of polygenic scores into healthcare systems facilitates better informed decision making by healthcare providers, advocating for a transition from a generalised approach to a more tailored, personalised healthcare strategy. Given that individuals inherit genetic predispositions to common complex diseases, leveraging polygenic scores holds promise in clinical applications, particularly in disease prevention and the refinement of more precise polygenic scoring systems. Recently, we developed a multiple trait polygenic score for type 2 diabetes, which, compared to a single trait polygenic score, had an improved prediction accuracy—quantified by explained variance—of 34% [52]. One of the information traits included was the polygenic contribution from current medication use [52]. In addition to body mass index, current medication use was the trait, which exhibited the largest importance in the construction of the multiple trait polygenic score. Similarly, multiple trait polygenic scores for coronary artery disease and ischaemic stroke were recently developed based on several similar information traits, which also enhanced the prediction accuracy [53,54]. The improvement in risk stratification is obtained by leveraging correlated trait information, i.e., the degree to which two, or more, complex traits share genetic information. It is not the information traits per se, which are important, but the degree of shared genetic information among them. Given the many different types of traits current medication use was genetically correlated with, medication use is a useful genetic information source. Although multiple trait polygenic scores have shown increased predictive performances, they do not currently have the discriminative ability needed to be used clinically.

This study has a number of limitations, which need to be addressed. First, despite the information on medication use being obtained by trained nurses during interviews, the same drug may have been reported under different names, which may limit the accuracy of the analysis. Second, the definition of drug usage used by UKB also included supplementary vitamins. However, for many diseases, dietary supplements, such as vitamins, are regularly used in the pharmacological intervention in common diseases. Thus, the exclusion of supplementary vitamins might not capture an individual’s medication profile more accurately. There is a clear need for future studies utilising more objective measures of medication usage. For example, a recent study by Aguayo-Orozco et al. utilised more than 1.1 billion prescriptions from the Danish prescription registry, aiming to model the risk of sequentially redeeming one drug after another [55]. Although this approach provides unprecedented insight into the prescription trajectories, it does not provide insight into the genetic predisposition towards medication use. It is imperative to utilise objective measures of any complex phenotypes when conducting genetic studies; equally importantly, as cross-study phenotypic heterogeneity attenuates statistical power and predictive ability [47,48], there is a need to better align phenotypic definitions, such as medication use, across studies. Third, the lack of information on medication duration, dosage and response means that true pharmacogenomic analysis cannot be performed. Similarly, this also limits the possibility of studying specific genetic alternations within the cytochrome P450 metabolising enzymes. Fourth, the assessment of current medication usage relied on self-reported questionnaires, potentially subject to individual interpretation and influenced by personal viewpoints. Such subjectivity could impact the accuracy of the gathered data. A previous study highlighted significant genetic correlations between self-reported diseases and medically diagnosed conditions [56], pointing to the fact that what people report in questionnaire data reflects their current health status. Similar findings have been reported for medication usage [57]. However, the responses are limited by potential recall bias, influencing the accuracy of participants’ recollection of past events or experiences. Moreover, the well-known healthy-volunteer bias, which is inherent to the UKB resource, does call for caution when interpreting the results obtained from UKB [58]. Fifth, the findings presented here are specific to UK Biobank participants, who are not representative of the general UK population [59,60] and may not translate to other populations and other health systems. In general, the majority of participants in existing GWASs are of European descent, despite the European population only accounting for 16% of the global population [61]. This constitutes a great inequity challenge, as it limits the discovery of novel causal genetic variants, which are exclusive to certain populations due to genetic drift [62]. Moreover, because the discovery GWASs lack ancestral diversity, the polygenic scores translate into poor generalisability across diverse ancestries and cohorts [63]. Many global efforts are currently under way to expand the diversity of GWAS data, which are further supported by the development of new statistical methods for improving the accuracy of PGS across populations by leveraging cross-population LD panels.

In conclusion, it was demonstrated that the genetic basis of current medication use in the UK Biobank among 335,744 individuals appeared genetically heterogeneous. A total of 59 independent quantitative trait loci for medication use were identified, and 18% of the observed variation could be ascribed to common genetic variants. The genetically heterogenous nature of medication use was further supported, as the genetic variance was spread across the genome, and the highest prediction accuracy was observed when 1.5 million genetic markers were included. Understanding the genetic aetiology of complex diseases has been suggested as a route for improving medical treatment. The majority of genetic variation within the human genome contributes to a large number of different complex traits and diseases. Therefore, incorporating correlated trait information into the polygenic score can increase the accuracy of risk stratification. Medication use—as defined in the current study—is an easily quantifiable trait, and due to its genetically corelated nature with many complex traits and diseases, leveraging such information into new multiple trait polygenic scores could further increase the accuracy of predicting disease predispositions and disease trajectories. Based on genetic data, individuals with high medication use can be identified; concurrently, these are the most diseased individuals, and they are at an increased risk for adverse drug reaction. Thus, individual medication profiles are likely to be yet another puzzle piece for understanding complex human diseases and for providing better medical treatment for the future generation.

## Data Availability

The UK Biobank individual genetic and phenotypic data were obtained from the UK Biobank (Application Number 31269), and a full list of the variables are available online. These data cannot be shared publicly due to the violation of patient privacy and the absence of informed consent for data sharing. The source code for this study is available upon request from the corresponding authors.

## Supplementary Materials

The following supporting information can be downloaded at: https://www.mdpi.com/article/10.3390/jpm14030319/s1, Supplementary File S1 contains Supplementary Figures S1–S8 and Supplementary Tables S4 and S5, whereas Supplementary Tables S1–S3 are available as Excel files. Figure S1. Distribution of number of medications used; Figure S2. Prediction accuracy of medication-use; Figure S3. Number of SNPs used in constructing genetic scores; Figure S4. Regression coefficient by percentiles of genetic scores; Figure S5. ICD10 diagnoses stratified by genetic scores; Figure S6. Number of individuals within top 50 ICD10 diagnoses; Figure S7. Medication-use within individuals with adverse drug reactions; Figure S8. Estimated genetic correlations to other medication traits; Table S1. List of ICD10 codes for adverse drug reactions; Table S2. Genome-wide significant loci for medication-use; Table S3. Estimated genetic correlations; Table S4. Medication-use among individuals with adverse drug reactions; Table S5. Estimated genetic correlations to other medication traits.
