# Supplementary material for: A Genetic Analysis of Current Medication Use in the UK Biobank
Source: J Pers Med. 2024 Mar 20;14(3):319. doi: 10.3390/jpm14030319 (PMC10971627; doi:10.3390/jpm14030319)
Supplement: Supplementary file 1 [file jpm-14-00319-s001.zip › Supplementary File S1.pdf]

Supplementary material for

A genetic analysis of current medication-use in the UK Biobank

Palle Duun Rohde

Genomic Medicine, Department of Health Science and Technology, Aalborg University, Denmark.

The supplementary material contains the following:

Supplementary Figures

Figure S1	Distribution of number of medications used.
Figure S2	Prediction accuracy of medication-use.
Figure S3	Number of SNPs used in constructing genetic scores.
Figure S4	Regression coefficient by percentiles of genetic scores.
Figure S5	ICD10 diagnoses stratified by genetic scores.
Figure S6	Number of individuals within top 50 ICD10 diagnoses.
Figure S7	Medication-use within individuals with adverse drug reactions.
Figure S8	Estimated genetic correlations to other medication traits.

Supplementary Tables

Table S1	List of ICD10 codes for adverse drug reactions. [EXCEL]
Table S2	Genome-wide significant loci for medication-use. [EXCEL]
Table S3	Estimated genetic correlations. [EXCEL]
Table S4	Medication-use among individuals with adverse drug reactions.
Table S5	Estimated genetic correlations to other medication traits.

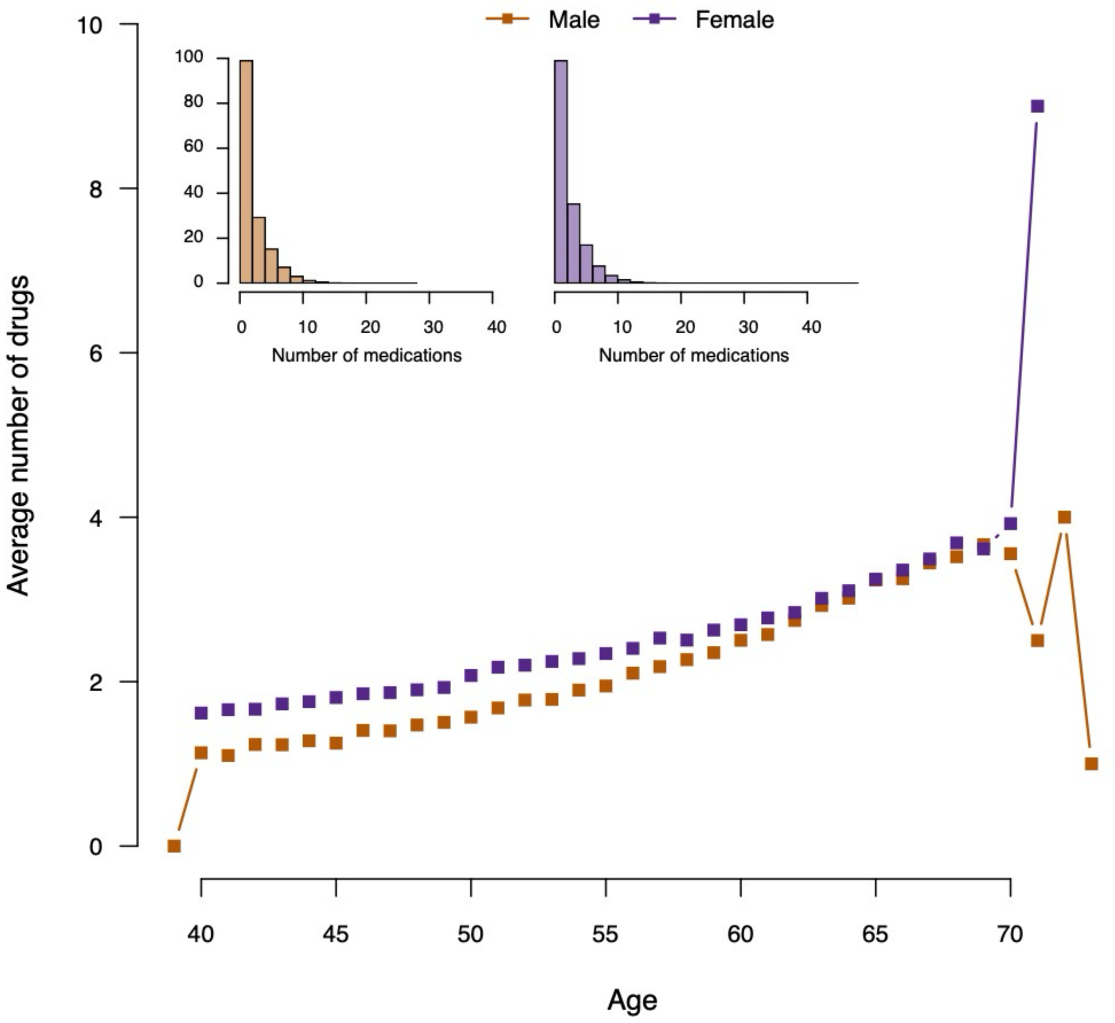

Figure S1 | Distribution of number of different drugs each individual in the UKB White British cohort stratified by age and sex.

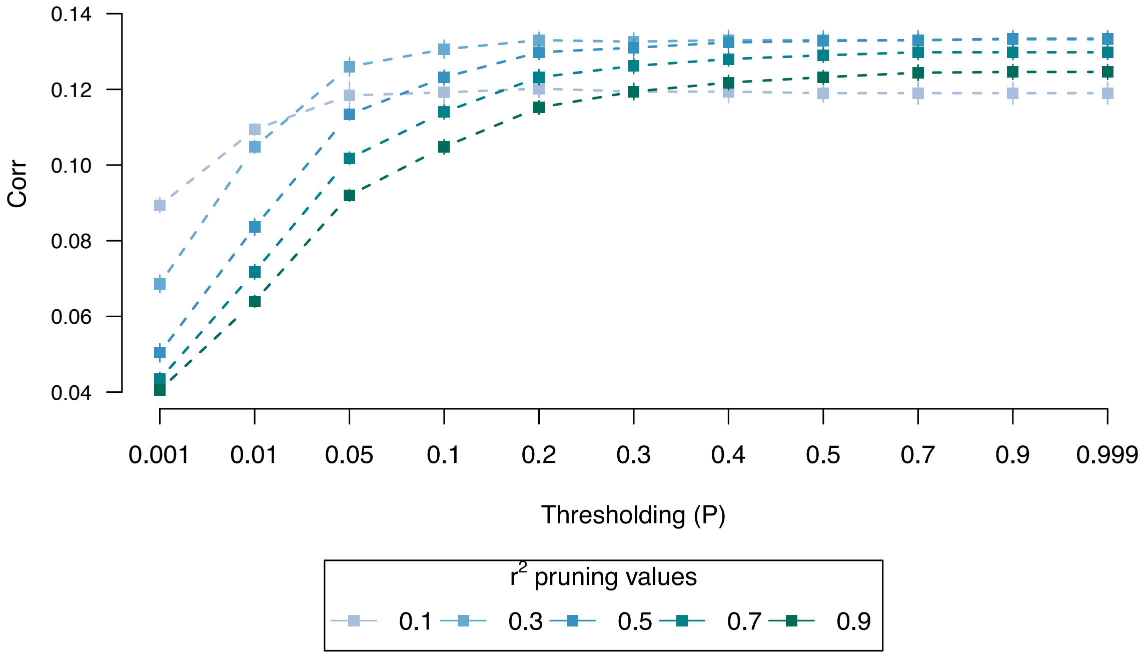

Figure S2 | Prediction accuracy (correlation between observed number of medications and the polygenic score in the validation data) for different pruning parameters.

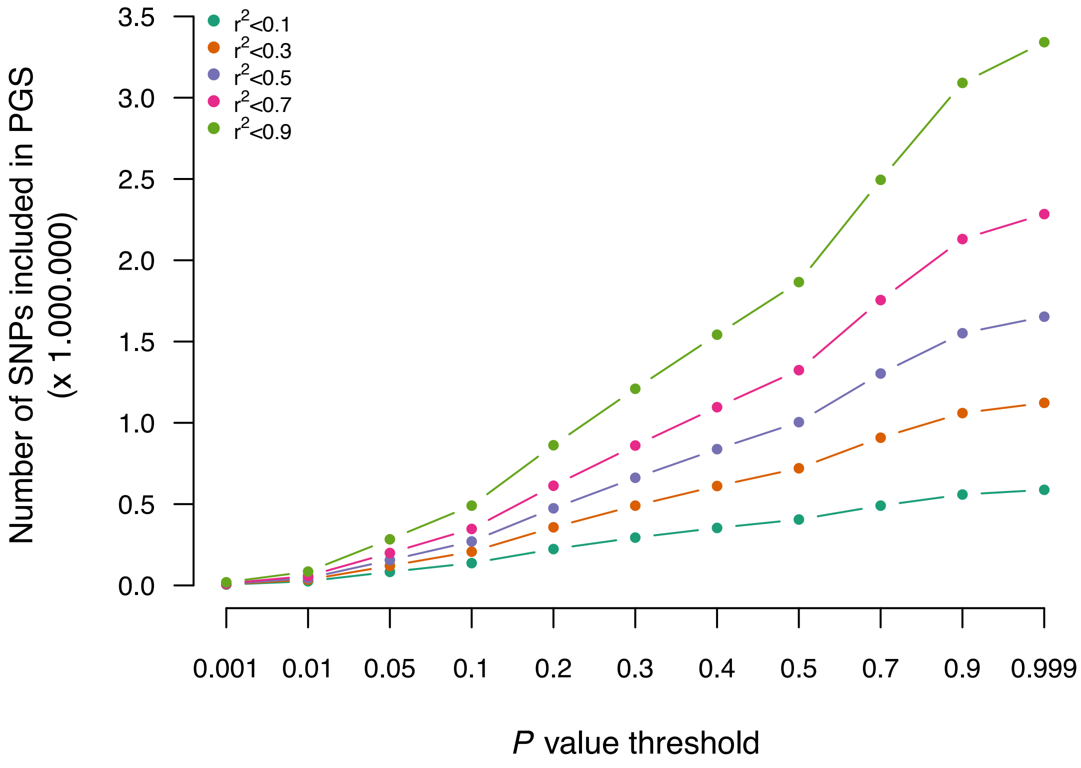

Figure S3 | Number of SNPs left after the different parameters for thresholding and LD pruning.

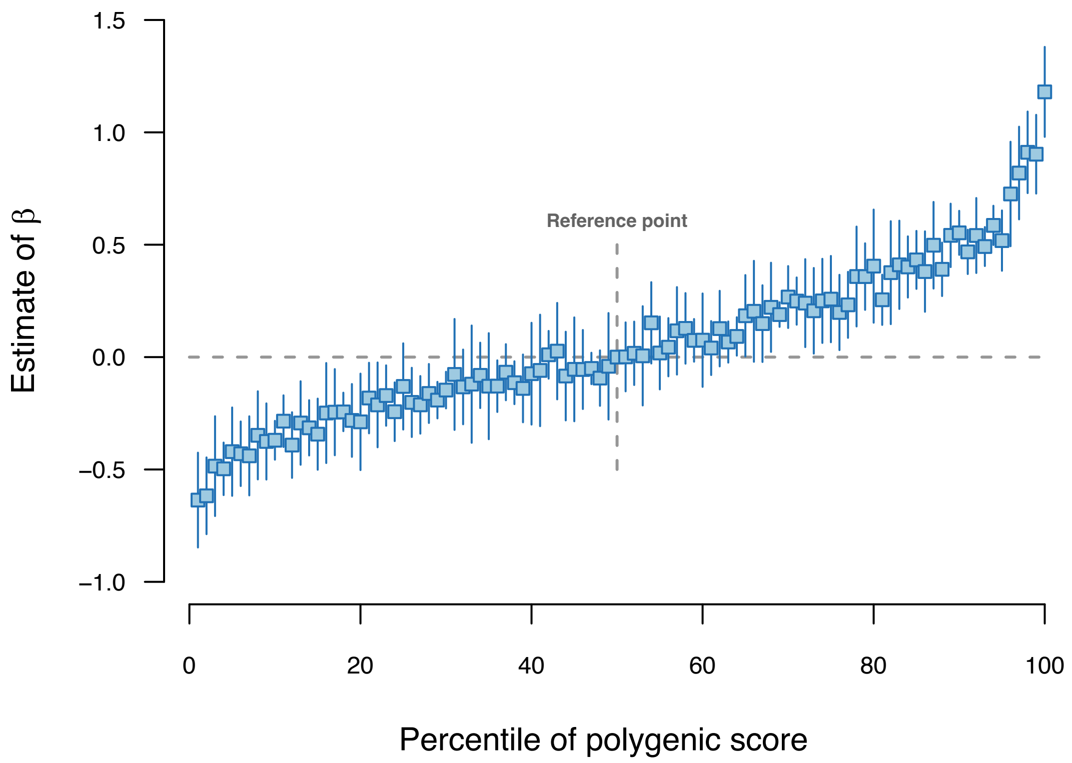

Figure S4 | Regression coefficient (β) by percentiles of polygenic scores. The β-estimates were obtained by regressing the number of medications on the polygenic score percentile relative to the 50th percentile adjusted for covariates. Results shown are for $r^2<0.5$ and is the mean (and 95% confidence interval) across the five training sets.

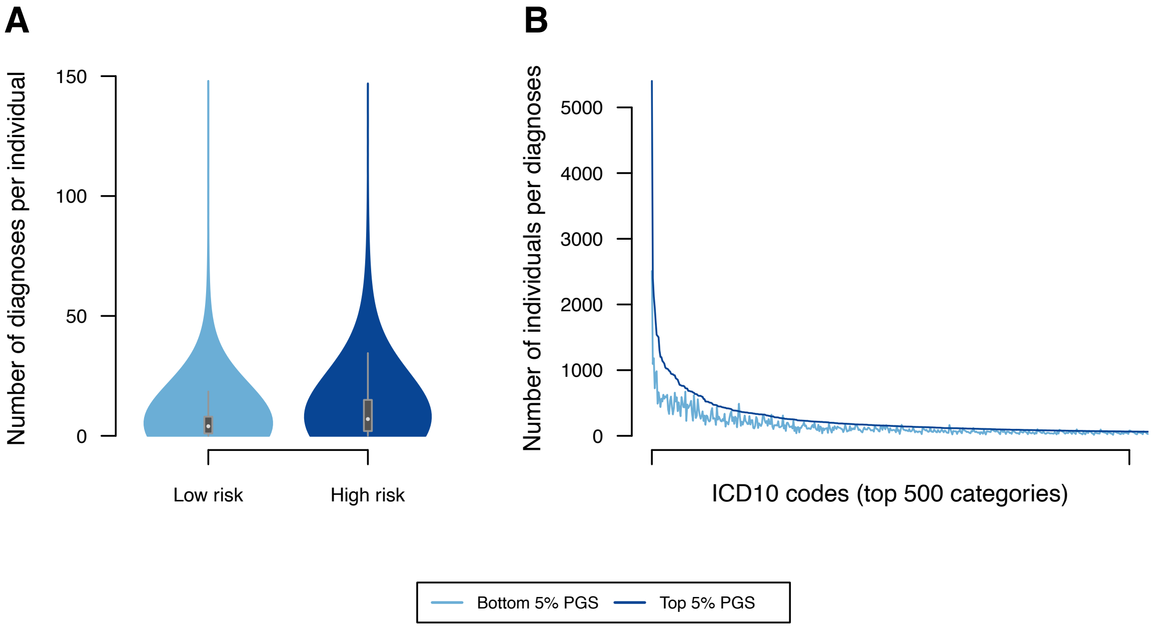

Figure S5 | **A**: Number of ICD10 diagnoses per individual within the bottom 5% (low risk) and in the top 5% (high risk) of polygenic scores. **B**: Number of individuals within each ICD10 diagnoses stratified by low and high-risk polygenic score. Only the 500 ICD10 codes with most cases are shown.

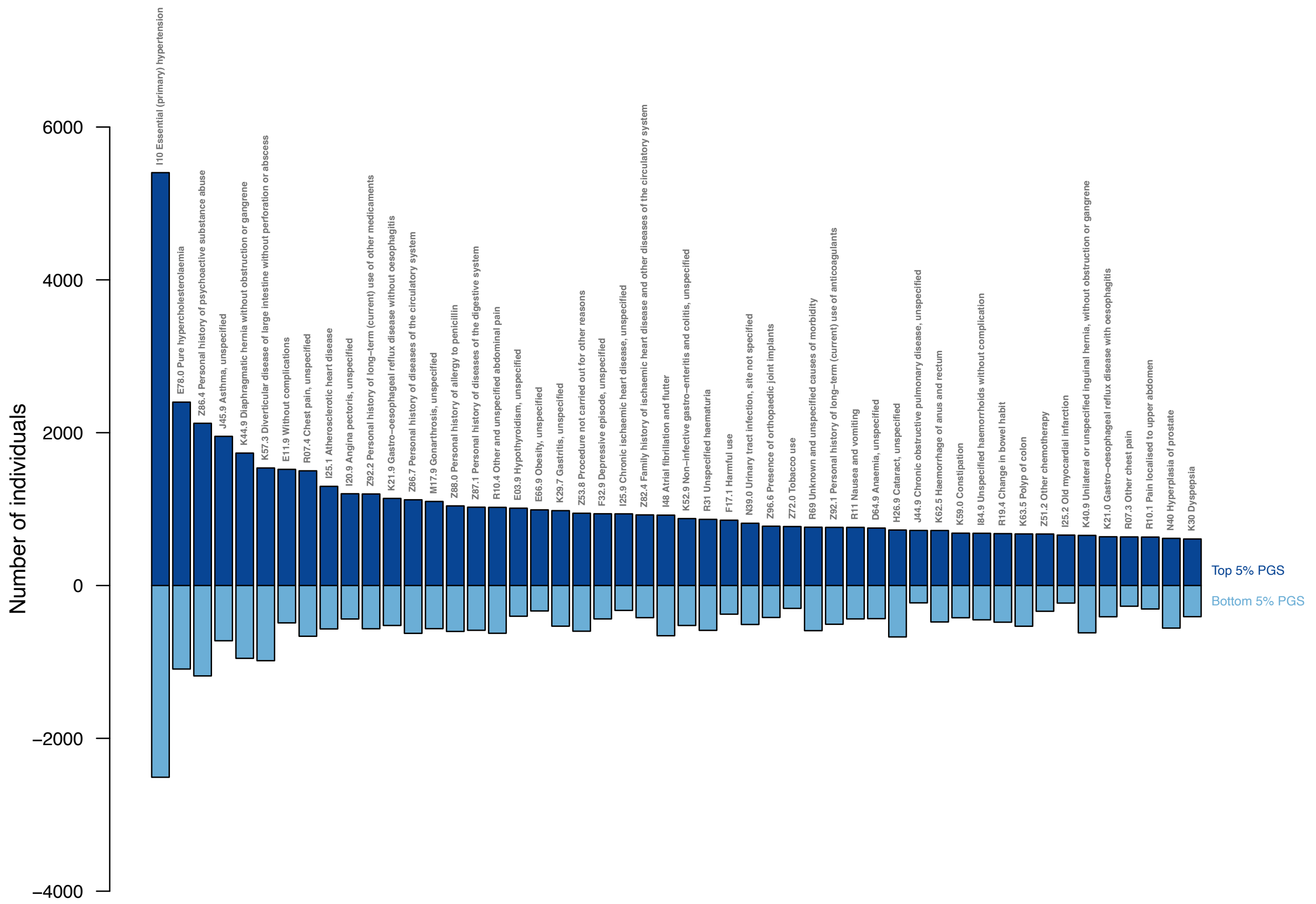

Figure S6 | Number of individuals within the top 50 ICD10 diagnoses codes of individuals with the top 5% highest polygenic score and bottom 5% lowest polygenic score.

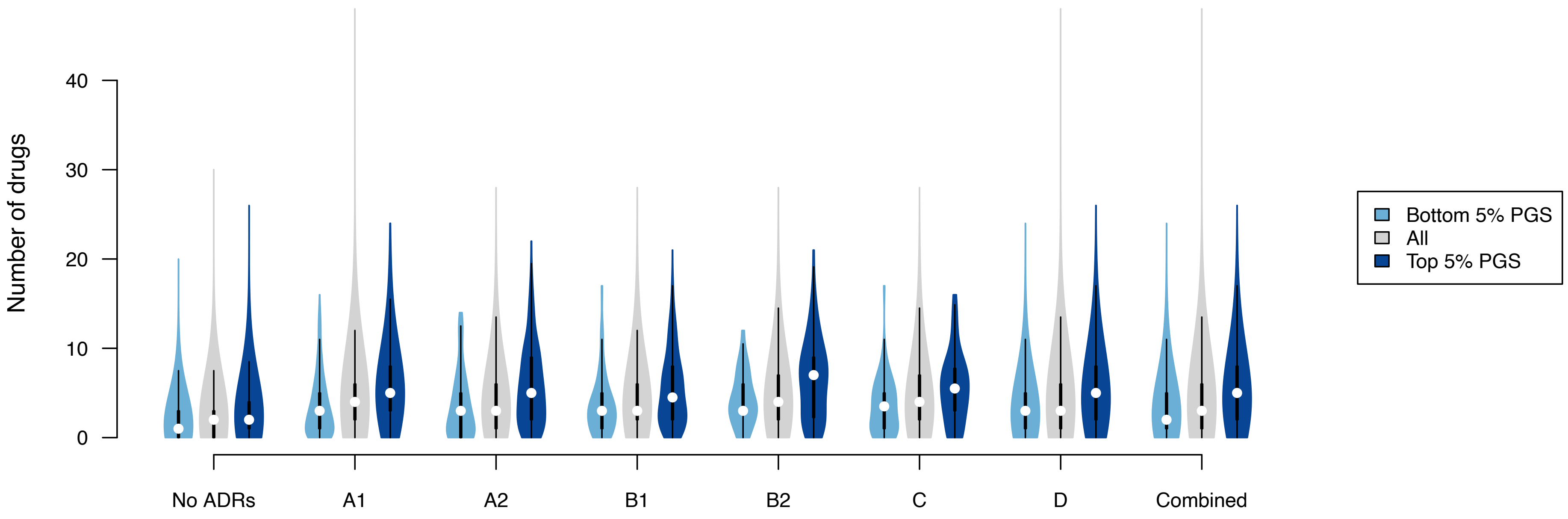

Figure S7 | Comparison of number of medications taken by individuals without an experience of any adverse drug reactions (ADR) and individuals suffering from at least one SDR stratified by low and high polygenic score. The classification of ADRs are based on Hohl et al (2014; doi:10.1136/amiajnl-2013-002116): A1: The ICD-10 code description includes the phrase 'induced by medication/drug'; A2: The ICD-10 code description includes the phrase 'induced by medication or other causes'; B1: The ICD-10 code description includes the phrase 'poisoning by medication; B2: The ICD-10 code description includes the phrase 'poisoning by or harmful use of medication or other causes'; D: Adverse drug event deemed to be very likely although the ICD-10 code description does not refer to a drug, and D: Adverse drug event deemed to be likely although the ICD-10 code description does not refer to a drug.

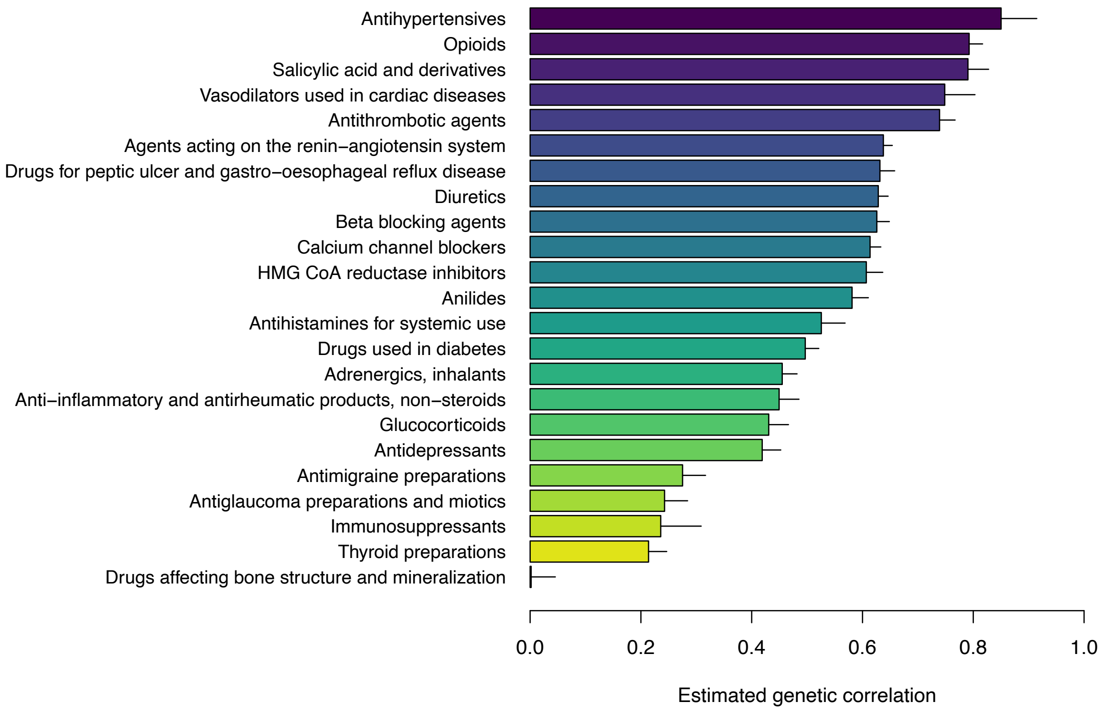

Figure S8 | Estimated genetic correlations between medication-use and the 23 categories of medications from Wu et al (2019, doi: 10.1038/s41467-019-09572-5). All estimates are significant, except 'Drugs affecting bone structure and mineralization'. See Supplementary Table S5 for details.

Table S4 | Comparison of number of drugs taken for individuals with or without adverse drug reactions (ADRs) for individuals with low or high polygenic score for medication-use.

	Low Risk			High Risk			
**ADR category**	**Mean**	**SD**	**n**	**Mean**	**SD**	**n**	***P* value***
No ADRs	1.69	2.03	15107	2.96	2.88	13988	$< 2.2 \cdot 10^{-16}$
A1	3.43	3.37	325	5.83	4.28	527	$< 2.2 \cdot 10^{-16}$
A2	3.40	3.66	94	5.81	4.51	160	$1.6 \cdot 10^{-6}$
B1	3.64	3.34	107	5.45	4.17	242	$6.4 \cdot 10^{-6}$
B2	4.20	2.94	29	6.60	4.53	78	0.006
C	3.55	3.19	58	5.57	3.96	74	0.002
D	3.27	3.10	1371	5.34	4.12	2288	$< 2.2 \cdot 10^{-16}$
Combined ADRs†	3.24	3.11	1650	5.28	4.08	2774	$< 2.2 \cdot 10^{-16}$
Multiple ADRs‡	3.81	3.52	274	6.19	4.48	478	$5.6 \cdot 10^{-16}$

* adjusted for age and sex

† Having at least one ADR within one of the six ADR categories

‡ Having more than one ADR within one of the six ADR categories

Table S5 | Estimated genetic correlations between medication-use and the 23 categories of medications from Wu et al (2019, doi: 10.1038/s41467-019-09572-5).

**Code**	**Name**	**Genetic correlation**	**Standard error**	***P* value**
A02B	Drugs for peptic ulcer and gastro-oesophageal reflux disease	0.63	0.027	5.26E-125
A10	Drugs used in diabetes	0.45	0.024	4.09E-92
B01A	Antithrombotic agents	0.74	0.028	2.14E-152
C01D	Vasodilators used in cardiac diseases	0.75	0.055	9.17E-43
C02	Antihypertensives	0.85	0.064	9.59E-40
C03	Diuretics	0.63	0.018	7.90E-274
C07	Beta blocking agents	0.63	0.023	1.83E-168
C08	Calcium channel blockers	0.61	0.019	3.43E-219
C09	Agents acting on the renin-angiotensin system	0.64	0.016	0.00E+00
C10AA	HMG CoA reductase inhibitors	0.61	0.030	1.03E-92
H03A	Thyroid preparations	0.21	0.033	6.89E-11
L04	Immunosuppressants	0.24	0.073	0.0012
M01A	Anti-inflammatory and antirheumatic products, non-steroids	0.45	0.036	5.57E-36
M05B	Drugs affecting bone structure and mineralization	0.002	0.044	0.9674
N02A	Opioids	0.79	0.024	8.85E-233
N02BA	Salicylic acid and derivatives	0.79	0.037	6.34E-100
N02BE	Anilides	0.58	0.029	2.98E-87
N02C	Antimigraine preparations	0.28	0.042	3.51E-11
N06A	Antidepressants	0.42	0.034	6.28E-36
R03A	Adrenergics, inhalants	0.46	0.027	1.16E-64
R03BA	Glucocorticoids	0.43	0.036	1.05E-33
R06A	Antihistamines for systemic use	0.53	0.043	1.74E-34
S01E	Antiglaucoma preparations and miotics	0.24	0.042	5.22E-09
